# A novel method to evaluate salivary flow rates of head and neck cancer patients after radiotherapy: a pilot study^☆^

**DOI:** 10.1016/j.bjorl.2017.03.004

**Published:** 2017-03-25

**Authors:** Luiz Felipe Palma, Fernanda Aurora Stabile Gonnelli, Marcelo Marcucci, Adelmo José Giordani, Rodrigo Souza Dias, Roberto Araújo Segreto, Helena Regina Comodo Segreto

**Affiliations:** a Universidade Federal de São Paulo (UNIFESP), Departamento de Diagnóstico por Imagem, Setor de Radioterapia, São Paulo, SP, Brazil; b Hospital Heliópolis, Serviço de Estomatologia e Cirurgia Bucomaxilofacial, São Paulo, SP, Brazil

**Keywords:** Head and neck neoplasms, Xerostomia, Radiotherapy, Saliva, Neoplasias de cabeça e pescoço, Xerostomia, Radioterapia, Saliva

## Abstract

**Introduction:**

The procedure used to evaluate salivary flow rate is called sialometry. It can be performed through several techniques, but none appears to be really efficient for post-radiotherapy patients.

**Objective:**

To adequate sialometry tests for head and neck cancer patients submitted to radiotherapy.

**Methods:**

22 xerostomic patients post-radiotherapy (total radiation dose ranging from 60 to 70 Gy) were included in this study. Ten patients were evaluated using sialometries originally proposed by the Radiation Therapy Oncology Group and twelve were assessed by our modified methods. Unstimulated and stimulated sialometries were performed and the results were classified according a grading scale and compared between both groups.

**Results:**

There was no statistically significant difference between the salivary evaluations of both groups (*p* = 0.4487 and *p* = 0.5615). Also, most of these rates were classified as very low and low.

**Conclusion:**

This novel method seems to be suitable for patients submitted to radiotherapy.

## Resumo

**Introdução:**

O procedimento utilizado para avaliar a taxa de fluxo salivar é denominado sialometria. Pode ser realizado por meio de várias técnicas, mas nenhuma parece ser realmente eficiente para pacientes pós-radioterapia.

**Objetivo:**

Adaptar sialometrias para pacientes com câncer de cabeça e pescoço submetidos à radioterapia.

**Método:**

22 pacientes xerostômicos pós-radioterapia (dose de radiação total variando de 60-70 Gy) foram incluídos neste estudo. Dez pacientes foram avaliados utilizando sialometrias originalmente propostas pelo *Radiation Therapy Oncology Group* e doze foram avaliados por nossos métodos modificados. Sialometrias não estimuladas e estimuladas foram conduzidas e os resultados foram classificados de acordo com uma escala de graduação e comparados entre os dois grupos.

**Resultados:**

Não houve diferença estatisticamente significante entre as avaliações salivares de ambos os grupos (*p* = 0,4487 e *p* = 0,5615). Além disso, a maioria dessas taxas foi classificada como muito baixa e baixa.

**Conclusão:**

Esse novo método parece ser adequado para pacientes submetidos à radioterapia.

## Introduction

The treatment for head and neck cancer (HNC) is based on three therapeutic modalities: radiotherapy (RT), chemotherapy, and surgery.^1^ The aim of RT is to control tumours with the least possible damage to adjacent normal tissues.^2^ For most initial cases, RT as a sole modality is considered the standard treatment; however, advanced cases must receive RT in association with chemotherapy and/or surgery.^3^

Despite efforts on RT planning to preserve non-neoplastic tissues in tumour region, these are inevitably included into the irradiation fields and suffer consequences as well.^4^, ^5^ Intensity and extent of the radiation-induced effects depends mainly on factors related to treatment such as total radiation dose, radiation dose per fraction, irradiated volume, dose distribution in tissue volume, association with chemotherapy,^2^, ^6^ and its duration.^7^

Regarding HNC treatment, the major salivary glands often receive significant radiation doses.^8^ Although the cytotoxic mechanisms of the radiation in salivary tissue are still not elucidated,^7^, ^9^ atrophy and acinar degeneration are histological findings often encountered.^10^, ^11^ As consequences, the subjective perception of dry mouth (or xerostomia) and the objective reduction in salivary flow rate (SFR) (or hyposalivation) are common,^7^, ^12^ dose-dependent, irreversible complications.^9^, ^13^ Additionally, they are almost always accompanied by changes in the salivary characteristics such as pH values, immunoglobulin levels, electrolyte balance, protein concentrations, viscosity, and colour.^8^, ^10^, ^12^

It is known that there is no direct relationship between xerostomia and low SFR, so efforts are needed to measure each one independently.^14^ In order to assess xerostomia in irradiated patients, some specific scales have been developed.^9^, ^13^ Likewise, quality of life questionnaires have the goal of evaluating xerostomia in conjunction with other well-described side effects of the HNC treatment.^13^, ^15^ Thus, even subjectively, an overview of the patient's state is achieved.

The procedure used to assess objectively SFR (sialometry) is performed using several techniques, each one having its own advantages, disadvantages, and challenges.^16^, ^17^ A poor reproducibility^16^ and a number of methods present in the literature,^4^, ^6^, ^10^, ^11^, ^13^ however, may lead to inconsistent results and inappropriate direct comparisons. Furthermore, there is no reliable, validated method to evaluate SFRs of HNC patients submitted to RT.

Based on the widespread 97-09 protocol of the Radiation Therapy Oncology Group (RTOG),^18^ sialometry tests were developed and applied in order to carry out easy, rapid, accurate assessments in HNC patients post-RT.

## Methods

### Patients and ethical considerations

A prospective study with 22 patients was conducted in the Division of Radiotherapy of the Universidade Federal de São Paulo (UNIFESP).

All the patients reported persistent xerostomia after megavoltage RT (3D planning) for HNC, with radiation fields encompassing major salivary glands (cervicofacial regions and supraclavicular fossae) and total radiation doses ranging from 50 to 70 Gy. Also, they were aged ≥18 years and had received the last RT session in a period from 3 to 36 months before the beginning of this study.

The present study was approved by the Research Ethics Committee of UNIFESP (protocols 0844/10-32449414.4.0000.5505) and all research subjects read and signed the informed consent form.

### Groups and general instructions

The patients were divided into two groups. Control Group consisted of 10 patients and Test Group of 12 patients. Control Group was assessed using the RTOG's 97-09 protocol^18^ and Test Group was evaluated by an adapted method developed by the authors.

All patients were evaluated at the same morning period by a dentist and were advised to stay at least 2 h without eating, drinking, smoking, and brushing their teeth. Also, during saliva collection they remained seated, with their eyes opened, and heads slightly bent forward.

### Modified sialometry tests (saliva collection)

**Figure 1:**
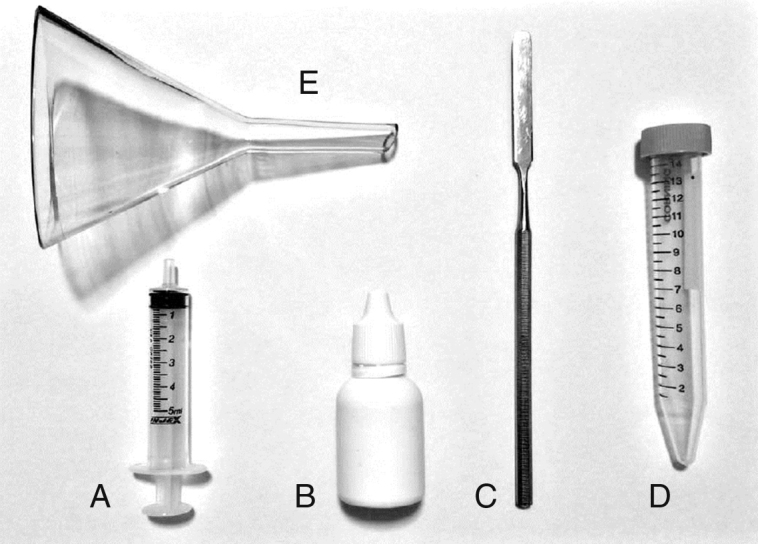
Material. (A) Syringe for measuring distilled water; (B) simethicone; (C) metal spatula; (D) tube graded in millilitres; (E) laboratory glass funnel.

Unstimulated sialometry: just before the saliva collection, the patients emptied their mouths of any saliva or mucous. After that, they accumulated saliva on the floor of the mouth, without swallowing, for 60 s. Then, they expectorated the accumulated saliva into a tube graded in millilitres (mL) with the aid of a laboratory glass funnel. It was repeated 4 more times for a total of 5 min. Next, a metal spatula and 2.0 mL of distilled water were used to remove the saliva adhered to the surface of the funnel. Also, 0.33 mL of simethicone (75 mg/mL) was added to the solution to eliminate gas bubbles and foamy saliva. Lastly, the tube was well shaken, the volume of saliva was measured, and the SFR per minute could be calculated. Some materials are shown in Fig. 1.

Stimulated sialometry: firstly, the patients emptied their mouths of any saliva or mucous. After that, 2% citrate solution was applied to the dorsolateral borders of the tongue, with a cotton tipped applicator, 5 times over 2 min (0, 30, 60, 90, and 120 s). Next, all the retained citrate solution in the mouth was eliminated. The steps of saliva collection and SFR assessment were the same as for the unstimulated sialometry.

### Data analysis

Descriptive analysis was used to summarize data on the patients, tumours, treatments, and sialometries. In addition, the unstimulated and stimulated SFRs, respectively, could be classified as: very low (<0.1 and <0.7 mL/min), low (0.1–0.25 and 0.7–1.0 mL/min), and normal (>0.25 to >1.0 mL/min).^17^

The mean sialometry values were also submitted to the Student's *t*-test for comparisons between both groups. The *p*-value was set at ≤0.05 to reach statistical significance.

## Results

### General

The patients’ age of Control Group ranged from 37 to 68 years (mean value: 56.3) and the patients’ age of Test Group ranged from 48 to 73 years (mean value: 61.75). Additional demographic features of the sample are summarized in Table 1.

**Table 1:** Patients’ demographic features.

Demographic features	Control group		Test group	
	Patients	%	Patients	%
*Gender*				
Male	9	90	7	58.3
Female	1	10	5	41.7

*Ethnic group*				
White	7	70	9	75
Black	3	30	3	25

*Alcohol consumption*				
Non-existent	2	20	0	0
Previous	7	70	8	66.7
Current	1	10	4	33.3

*Tobacco consumption*				
Non-existent	1	10	0	0
Previous	7	70	10	83.3
Current	2	20	2	16.7

Features of the tumours and treatments are described in Table 2.

**Table 2:** Features of the tumours and treatments.

Features	Control group		Test group	
	Patients	%	Patients	%
*Histological type of the tumour*				
Squamous cell carcinoma	9	90	12	100
Adenoid cystic carcinoma	1	10	0	0

*Primary site of the tumour*				
Oral cavity	0	0	2	16.6
Pharynx	8	80	5	41.7
Larynx	2	20	5	41.7

*Stage of the tumour*				
I	1	10	2	16.7
II	0	0	1	8.3
III	0	0	1	8.3
IV	9	90	8	66.7

*Surgery*				
Yes	4	40	6	50
No	6	60	6	50

*Total radiation dose*				
60–69 Gy	3	30	6	50
70 Gy	7	70	6	50

*Chemotherapy*				
Yes	10	100	10	83.3
No	0	0	2	16.7

### Saliva sampling

**Figure 2:**
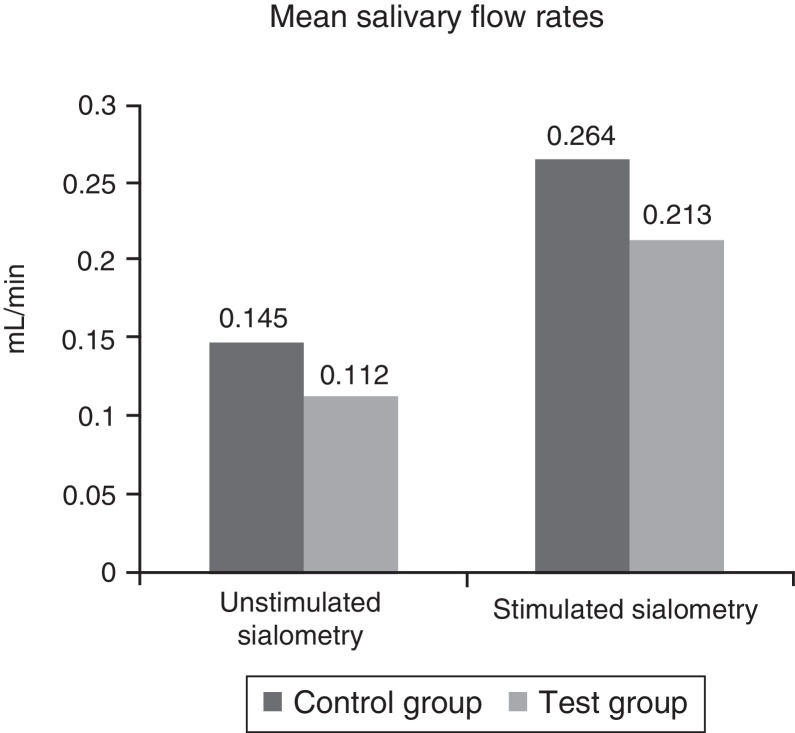
Mean salivary flow rates. The mean salivary flow rates of both groups, in millilitres per minute (mL/min). No statistically significant difference was obtained (*p* = 0.4487, *p* = 0.5615).

The averages of the unstimulated and stimulated SFRs are summarized in Fig. 2 and their classifications in Table 3. Additionally, there were no statistically significant differences between both groups (*p* = 0.4487 and *p* = 0.5615).

**Table 3:** Classification system of the salivary flow rates.

Salivary flow rate	Control group		Test group	
	Patients	%	Patients	%
*Unstimulated*				
Very low (<0.1 mL/min)	3	30	5	41.7
Low (0.1–0.25 mL/min)	6	60	5	41.7
Normal (>0.25 mL/min)	1	10	2	16.6

*Stimulated*				
Very low (<0.7 mL/min)	10	100	11	91.7
Low (0.7–1.0 mL/min)	0	0	1	8.3
Normal (>1 mL/min)	0	0	0	0

Regarding unstimulated sialometries, the salivary flow rates of both groups ranged from 0 to 0.3 mL/min. The median of Control Group was 0.16 mL/min and the standard error was 0.0296 mL/min. The median of Test Group was 0.1 mL/min and the standard error was 0.0307 mL/min.

Concerning stimulated sialometries, the SFR of Control Group varied from 0.04 to 0.5 mL/min, with the median 0.2750 mL/min and the standard error 0.0494 mL/min. The SFR of Test Group ranged from 0 to 0.8 mL/min, with the median 0.1650 mL/min and the standard error 0.0665 mL/min.

## Discussion

Sialometries are performed by drainage, expectoration, or weighing cotton wool balls soaked with saliva. Some of these techniques aim to selectively collect the secretion of each salivary gland, but with little clinical applicability (e.g. catheterization of salivary ducts). On the other hand, techniques which take into account the whole saliva volume collected over a period of time are the most used since they are faster, easier, and cheaper.^16^

The well-known RTOG's protocol has been developed to evaluate the mitigating effect of pilocarpine on hyposalivation and mucositis in patients undergoing RT.^18^ So, from our experience in using this protocol for irradiated patients with no method of prevention and treatment for hyposalivation,^19^, ^20^ we considered necessary adapting it to post-RT patients. The unstimulated sialometry, in particular, could be substantially improved by our methods, since it is based on the collection of extremely small amount of saliva.

During the procedures, the glass funnel facilitated the saliva collection and also prevented a possible volume loss due to the larger area for expectoration. The highly viscous saliva that adhered to the funnel surface could be easily removed with the aid of the metal spatula and distilled water. Another important point was the addition of simethicone to the solution to decrease the surface tension of gas bubbles and to disperse foam. Thus, we could measure the total saliva volume immediately, avoiding further losses related to the need of leaving the saliva samples to rest.

Concerning the presence of stimulation in sialometries, gustatory (citric acid) and mechanical agents (paraffin, silicone, unflavoured chewing gum) are used to simulate patients’ conditions throughout the day (e.g. eating and chewing). It is believed that the absence of stimulation reflects the physiological status of the sublingual and submandibular glands, as these are responsible for baseline salivary secretion. On the other hand, mechanical stimulants promote marked response of the parotid glands and gustatory stimulants activate the three pairs of major salivary gland simultaneously.^17^

From our standpoint, clinicians should carry out both sialometries for a thorough evaluation of irradiated patients. For the stimulated sialometry, the use of 2% citrate solution seems to be more advantageous because the three pairs of major salivary glands (responsible for 90% of saliva output) are evaluated at the same time.^7^, ^12^ Also, edentulous patients cannot be assessed using mechanical stimulations.

Our data showed the marked, persistent, well-recognised radiation-induced reduction in SFR.^7^, ^9^, ^12^, ^13^, ^19^, ^20^ The lack of statistically significant difference between both groups and the quite similar results obtained suggest that our modifications in RTOG's protocol were satisfactory and applicable. In general, the results from the novel sialometries could be obtained more quickly than those of RTOG. Moreover, our method was easier than the others and did not require costly materials, factors really important for the routine clinical use.

## Conclusion

This paper encourages further researches with bigger samples to apply these novel sialometries in post-irradiated patients. Likewise, it would be interesting to investigate whether this method is suitable for other diseases and pathological conditions which also result in low SFR (e.g. Sjögren's Syndrome).

## Conflicts of interest

The authors declare no conflicts of interest.
